# Surface-Free Multi-Stroke Trajectory Reconstruction and Word Recognition Using an IMU-Enhanced Digital Pen

**DOI:** 10.3390/s22145347

**Published:** 2022-07-18

**Authors:** Mohamad Wehbi, Daniel Luge, Tim Hamann, Jens Barth, Peter Kaempf, Dario Zanca, Bjoern M. Eskofier

**Affiliations:** 1 Machine Learning and Data Analytics Lab., Department Artificial Intelligence in Biomedical Engineering, Friedrich-Alexander-Universität Erlangen-Nürnberg (FAU), 91052 Erlangen, Germany; daniel.luge@fau.de (D.L.); dario.zanca@fau.de (D.Z.); bjoern.eskofier@fau.de (B.M.E.); 2 STABILO International GmbH, 90562 Heroldsberg, Germany; tim.hamann@stabilo.com (T.H.); jens.barth@stabilo.com (J.B.); peter.kaempf@stabilo.com (P.K.)

**Keywords:** trajectory reconstruction, inertial measurement unit, sensor-based deep learning, convolutional neural network, handwriting recognition, digital pen

## Abstract

Efficient handwriting trajectory reconstruction (TR) requires specific writing surfaces for detecting movements of digital pens. Although several motion-based solutions have been developed to remove the necessity of writing surfaces, most of them are based on classical sensor fusion methods limited, by sensor error accumulation over time, to tracing only single strokes. In this work, we present an approach to map the movements of an IMU-enhanced digital pen to relative displacement data. Training data is collected by means of a tablet. We propose several pre-processing and data-preparation methods to synchronize data between the pen and the tablet, which are of different sampling rates, and train a convolutional neural network (CNN) to reconstruct multiple strokes without the need of writing segmentation or post-processing correction of the predicted trajectory. The proposed system learns the relative displacement of the pen tip over time from the recorded raw sensor data, achieving a normalized error rate of 0.176 relative to unit-scaled tablet ground truth (GT) trajectory. To test the effectiveness of the approach, we train a neural network for character recognition from the reconstructed trajectories, which achieved a character error rate of 19.51%. Finally, a joint model is implemented that makes use of both the IMU data and the generated trajectories, which outperforms the sensor-only-based recognition approach by 0.75%.

## 1. Introduction

Handwriting is an essential method to transfer and record information in daily lives. With the advancements in technologies, digitally recording data input by smart devices, e.g., text input without a keyboard, has become a popular application in the domain of human–computer interaction. In recent years, several systems have been developed for the application of handwriting recognition, in which text data is automatically recognized and digitized into a machine-form text, and have achieved notable recognition rates [1]. However, the application of such systems is limited to digitizing text, and cannot be used when the requirements extend beyond simple text recognition, such as visually seeing input text or drawn pictures, in which the reconstruction of the trajectory of movement is required.

Trajectory reconstruction (TR) is defined as the positional change estimation over time, and can be used to retrace the movements of an object in space. TR is used in several domains, including robotics and localization, and is often based on visual systems [2,3], requiring a controlled camera-based environment, with expensive equipment and high computational image-processing techniques, to function. Contrarily, inertial sensors are low cost and self-sustained, and, therefore, provide an advantageous setup for applications of TR.

Inertial-measurement-unit (IMU)-based systems have gained popularity throughout the previous years in several domains in which camera-based systems prevailed. Classification applications using IMUs, in which movements are classified into a set of classes (human activity [4], gesture [5,6,7], and handwriting [8,9] recognition), have been developed with adequate accuracies. However, TR applications using IMUs are still limited to large-scale applications, in which the accepted margin of error is not affected by sensor error accumulation over time, due to the large distances considered in the applications. Applications include autonomous vehicles localization [10,11], pedestrian tracking [12,13], and indoor localization [14,15], which track the movements of vehicles or subjects on a large-scale surface area.

However, solutions for small-scale TR, such as handwriting, are still limited. Handwriting TR requires precision on a millimeter level, narrowing the accepted error rates considerably in comparison to larger scale applications. Moreover, slight external disturbance that can occur during operation, which can usually be neglected in larger scale applications, can significantly affect the quality of handwriting reconstruction. The trembling of the hand while holding the pen is enough to hinder a gesture recognition system as ineffective for recovering handwriting movements.

Current available solutions for handwriting TR rely on the positional-based detection of writing, which constrains a system to multiple devices. A digital pen along with a supporting writing surface run simultaneously [16], from which a coordinate-based trajectory is reconstructed. However, several limitations occur when using such systems which often limit the capabilities of human–computer interaction in terms of usability. Primarily, the compatibility between digital pens and writing devices poses a limitation to freely using any pen on any device. Occasionally, technical difficulties occur that limit devices from detecting the pens writing on them due to pairing difficulties between the two devices. Secondly, the size of writing screens limits the capability to freely write in wide areas. Small writing screens cause instability of the writer’s hand, which, in turn, affects the speed and strength, and, consequently, the quality of writing. To tackle these limitations, IMUs can be used, integrated within writing devices, that allow tracking of the movements of a smart pen, without the need for other devices to track the position of a pen tip on a digital surface.

The usage of IMUs for handwriting TR poses difficulties due to errors occurring in the recorded data as a result of sensor drift. To address this issue, previous studies [17,18,19] providing IMU-based handwriting tracking solutions, mainly followed classical sensor fusion approaches, which require heavy pre-processing, and increased system complexity. Such systems required data segmentation, relative motion extraction, and displacement calculation of sensor data. Moreover, an initial pose estimation, coordinate transformation, as well as post-processing rotation, including slant and slope estimation and correction, were required to perform the TR task. However, when a large range of linear, rotational, and translational movements is considered, the obtained precision is limited to situation-specific adaptations of IMU sensors, which can be overcome by the means of a data-driven system using artificial neural networks.

**Figure 1:**
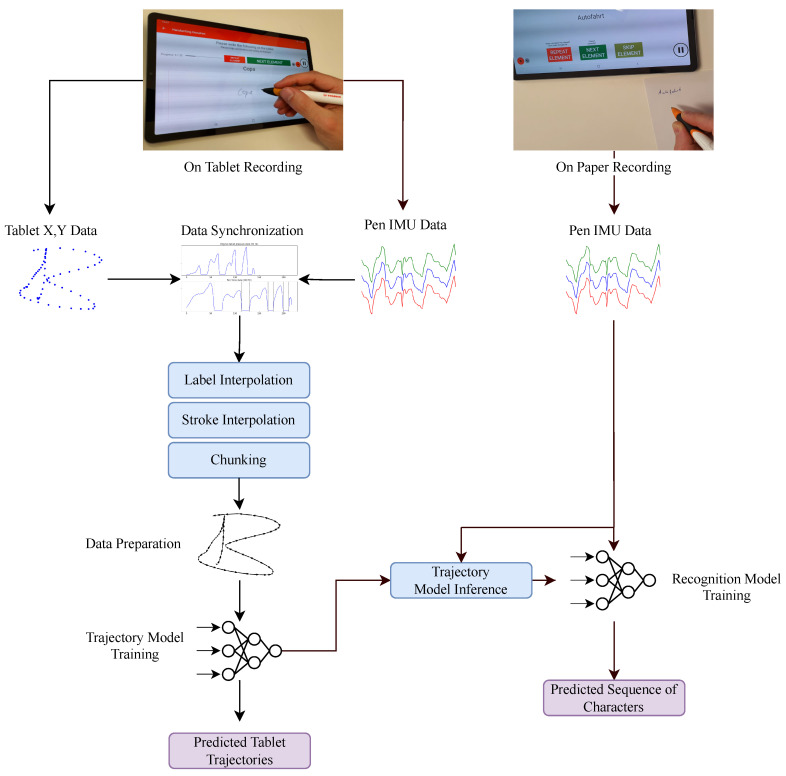
A visual summary of the workflow of our paper. We develop an end-to-end neural network model for handwriting trajectory reconstruction using data collected by writing with a digital pen on a tablet. The system was also tested on writing on paper by developing a text recognition model using IMU data and the relative generated trajectory data.

Neural networks have shown successful results for modeling recognition systems for handwriting; however, they have not been explored for the application of handwriting TR. In this paper, we aim to fill this gap by presenting a data-driven approach for small scale trajectory reconstruction using an IMU-enhanced digital pen that is surface-independent and requires no additional equipment or data segmentation in operation, but only for data collection and model training. In contrast to previous studies, our solution requires minimal pre-processing in terms of data segmentation and displacement calculation, but instead is based on convolutional neural networks (CNNs) that directly map raw IMU data into displacement data. A digital pen is used to write on a tablet, from which ground-truth coordinate data is collected relative to the movements of the pen. The data is then used to train a CNN to retrace the trajectories from the movements of the pen from the sensors’ data. The predicted trajectories are evaluated, afterwards, via distance measures in comparison to the collected ground-truth data. Additionally, we use data collected by writing on paper to validate the effectiveness of the system for writing on regular paper. A writing recognition system is developed using trajectories generated for data obtained by writing on paper, and is compared with the recognizer developed with the original IMU data. A graphical summary of the procedure in this work is shown in Figure 1.

The next chapters are outlined as follows. Section 2 presents a summary of the commercial and previous solutions provided for handwriting TR. In Section 3, the data-collection method, for the extraction of positional data from a tablet that relatively correlates to the IMU data recorded by the pen while writing, is presented with the different methods of data synchronization and data pre-processing. In the same chapter, we describe the architecture and implementation details of the neural network that allows the reconstruction of complete words. Section 4 presents the results obtained from our experiments of trajectory reconstruction and word recognition. Finally, Section 5 and Section 6 discuss the obtained results and conclude the paper.

## 2. Related Work

Handwriting digitization solutions have been developed in several studies and products throughout the past years, considering the importance of the application in topics such as picture drawing, note taking, and document signing [20,21,22]. Yet, even with the advancements in technology, the topic of digital-pen tracing lags behind such advancements, and still requires the use of multiple devices to acquire adequate pen movements on a surface. In this section, we discuss some of the available products and solutions in which pen reconstruction has been developed as an application, including prior research focusing on TR only via IMUs.

### 2.1. Commercial Products

Many commercial solutions have been introduced in the past decade that efficiently reconstruct written text into a digital form. Popular smart pens, such as Samsung Spen [23] or Apple Pencil [24], are supplied as accessories for mobile devices and their use is limited to this pairing. Other products are presented in the form of smart notebook sets [25,26,27], removing the restriction of writing on tablets and providing a more natural handwriting experience. In such solutions, data is processed in different ways varying from taking pictures of written documents, using grid-based paper, or using tablets behind papers to capture pen movements. Such solutions present accurate trajectory recovery, however, are positional-based and rely on specific writing surfaces to detect pen movements.

### 2.2. IMU-Based Recognition Systems

Several solutions have been developed throughout the past years for the application of handwriting recognition using inertial data. Character-level classification models for the Latin alphabet by [28,29,30,31] showed an accuracy of up to 83%, while word-recognition models showed recognition rates of 82.03% [32]. These systems presented recognition models, in which digitized text is output from the models, but no models for the trajectory reconstruction of the digital pens, where the output is the actual handwriting instead of digital text, were developed.

### 2.3. IMU-Based Trajectory Reconstruction Systems

In contrast to commercial products, limited studies have considered trajectory reconstruction using IMU-enhanced devices. *Gyropen* [17] used a mobile phone’s corner as a writing device, retracing an approximation of the movements of the phone via the angular velocity rotating around a fixed virtual center point. The system was used to reconstruct the Latin alphabet, with a recognition rate of 82% after reconstruction, yet was limited to single strokes. Similarly, noise reduction methods were studied in [18,33] using a smartphone to retrace the movements of the device, using angular velocity and linear acceleration, by calculating the displacement through data integration with a reset switch mechanism to compensate for accumulated integration errors. Finally, neural networks were used to classify trajectories into the lowercase single strokes Latin characters, achieving an accuracy of approximately 93% on 19 classes. An IMU-integrated pen was used in [34] to reconstuct single-stroke shapes, which achieved an accuracy of 90.4% for the classification of ten digits.

Recently, *Handwriting-Assistant* [19] introduced an attachable IMU-based pen cap, which measured the pen-tail linear acceleration and angular velocity throughout the writing process. Writing was divided into on and off plane, then segmented single strokes were reconstructed, rotated, and aligned to retrieve the complete reconstruction of the complete trajectory. The system achieved a normalized error rate between a minimum of 0.07 and maximum of 0.13, varying with the pen length. The system was also evaluated on single-character recognition of the Latin alphabet, achieving a recognition rate of 98.2%.

The described works presented different approaches of reconstructing movements using IMU data recorded on different devices. However, in terms of TR, the different systems reconstruct only single strokes, resulting in reconstructed cursive word writings with no pen-up between letters, and demonstrated effectiveness only on short pen traces. Additionally, different pre-processing steps, such as tip estimation and coordinate transformation, were required, as well as post-processing rotation and alignment steps being implemented to present the final reconstruction. Moreover, the systems were evaluated on the recognition of single characters, which, overall, included single or two strokes. Meanwhile, the solution presented in this paper allowed the reconstruction of complete words, using a neural network approach, without the need of segmentation or post-processing alignment during inference, and provided the recognition rates on complete words. Table 1 presents a summary of the different works discussed, showing the different aspects required for the functioning of the systems.

## 3. Materials and Methods

In this section, we introduce the tools and methods used to develop our system. We present the digital pen used for writing, the data-collection methods that allow the collection of labeled ground-truth data, and then present the CNN model that makes use of labeled data in a supervised learning approach to map sensor data to coordinate trajectory data.

### 3.1. Data Acquisition

**Figure 2:**
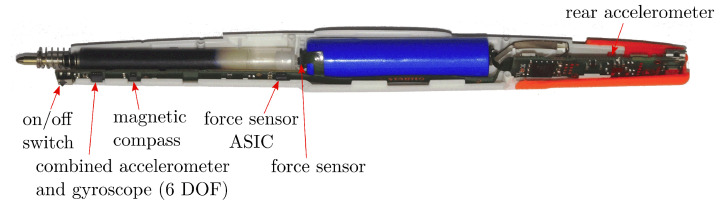
Sensor distribution in the Digipen.

The *STABILO Digipen* [35] is a regular ballpoint pen, equipped with multiple sensors, that allows writing on paper while acquiring sensor data in the process, and has been used in several works for the task of handwriting recognition [29,30,31,32]. It streams data via an integrated Bluetooth module at a rate of 100 Hz, and can be paired to a handheld device on which data with a total of 13 channels are collected along with timesteps of the recording time. Six channels include data from two triaxial accelerometers (front and back of the pen), adjusted to a range of ±2 g with a resolution of 16 Bit (front) and 14 Bit (rear). Three channels are for the gyroscope (3 axes) of a range of ±1000°/s with a resolution of 16 Bit, and three cover the magnetometer (3 axes) data that has a range of 2.4 mT with a resolution of 14 Bit. The final channel is for a force sensor that has a measurement range of 0 to 5.32 N with a resolution of 12 Bit, which helps in distinguishing when writing is taking place during the recording. Figure 2 shows the distribution of the sensors along the pen.

The Samsung Galaxy Tab S6 [36] was used for our data collection, which allows data recording at a rate of 60 Hz with a resolution of 2560 × 1600 pixels per inch. The data collected included the X, Y, and Z coordinates, along with the pressure with which the user was writing, in addition to recording time.

**Figure 3:**
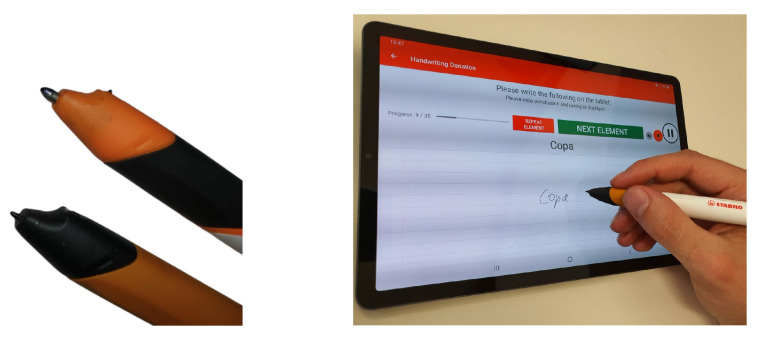
Wacom compatible writing tip on the Digipen (**left bottom pen**), and the recording app (**right**).

In order to acquire ground-truth data as coordinate data, the regular tip of the Digipen was replaced with a *Wacom* compatible electro-magnetic resonance tip, which changes the Digipen to the form of stylus pen, allowing writing on tablet, as shown in Figure 3 (left). A recording app provided by STABILO guided the user throughout the recording process, and collectd the IMU sensor data and tablet positional data. Additionally, the app instructed the user with the next word to be written, which allowed the collection of text labels, as shown in Figure 3 (right).

The data was collected with three Digipens to ensure that the system is not biased towards a single pen. Participants were seated in a regular writing environment and were requested to write the words that were displayed on a tablet, then switching between different labels manually upon completion. No constraints on the writing style was set, as both cursive and non-cursive writing were accepted for the study. A total of six users participated in the study. The users were of different ages, included five males and one female, were right handed, and were familiar with writing the Latin alphabet. Each recording session provided the total recording data separated between sensor data, tablet data, and word text labels.

### 3.2. Data Synchronization & Preparation

Synchronization between the pen data and tablet using the recorded timesteps was not a viable solution due to lagging problems that occured upon sending data from the pen to the tablet. Additionally, the different sensitivity between the pen-force sensor and the tablet-pressure sensor posed a limitation for using alignment algorithms, such as dynamic time wearping, to align both signals by the applied force. Finally, the different sampling rates between the systems hindered having time-distributed data points between the two systems. Time-distributed is defined in the rest of the paper as having a single IMU datapoint represented as a single coordinate datapoint.

In order to synchronize the data between the pen and the tablet, the word text labels were used to determine between which time steps a specific word was written in both systems. The lag between the two systems was removed by the hovering movements prior and subsequent to the writing carried out by the user when switching between the labels. To ensure adequate quality of data, all hovering data points before and after the recording of a word were discarded, while keeping hovering data points in between the start and end of writing, determined by the force sensor and pressure data, representing multiple strokes of a word. Here, a stroke is defined as a continuous touch or hover movement.

**Figure 4:**
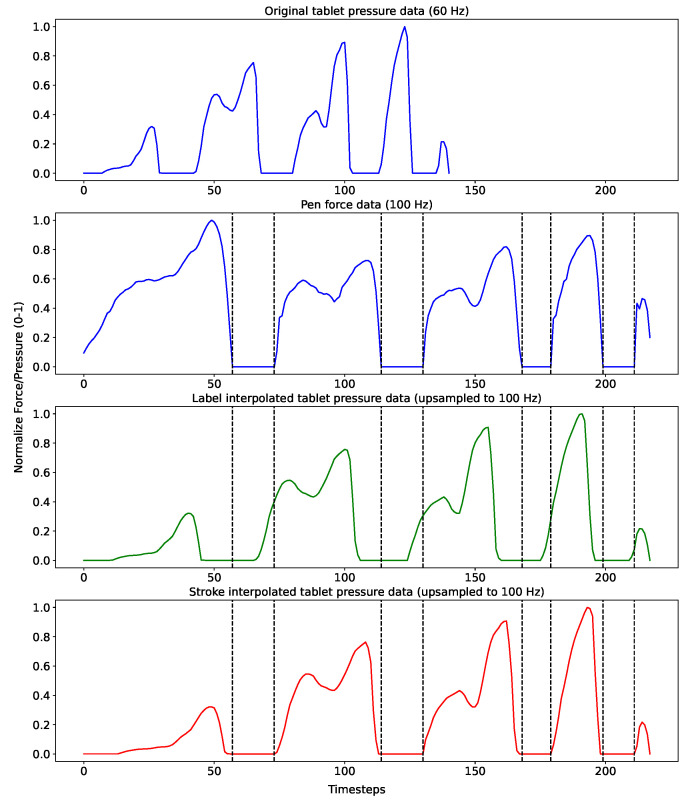
Tablet data upsampling via complete label interpolation and stroke-based interpolation.

In order to obtain signals with the same sampling rate for subsequent processing, tablet data was upsampled using linear interpolation to match the length of the relative sensor data for each word sample, which provided time-distributed data between the two systems. This is referred to in the rest of the paper as *‘Label’* interpolation. However, linearly interpolating the complete samples changed the representation of the applied pressure on the tablet data in comparison to the pen data, such that the force applied at different timesteps of the recording differ between the two. This caused the data to include touch datapoints on the tablet while hovering with the pen, and vice versa, as shown in Figure 4, which represents a normalized force and pressure plot of a randomly selected sample. To avoid having incorrectly aligned force distributions, each recording was split into different touch and hover strokes, and each tablet stroke was upsampled to the relative sensor stroke, which allowed to have equivalent forces at each timestep of the recording, hereon referred to as *’Stroke’* interpolation. Strokes were identified by the values recorded by the tablet-pressure sensors and pen-force sensor. Zero valued data represented hovering strokes, whereas non-zero valued data (irrelevant of amplitude) represented touch strokes in both the tablet and pen data.

**Figure 5:**
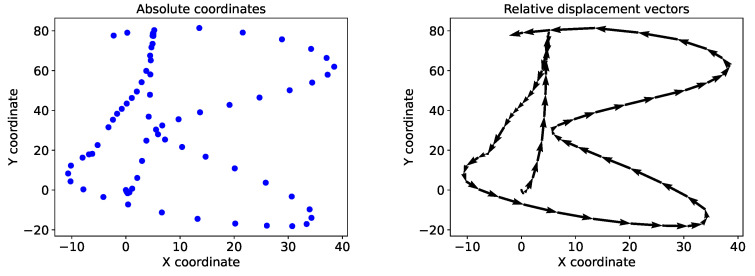
Absolute coordinates of the character *‘B’* (**left**), and the calculated relative displacement vectors of the same character (**right**).

The tablet recorded the absolute position of the pen tip on the screen at each timestep. However, since the aim of the system was to retrace the tip of the pen between a start and end position on a surface, the absolute positions in the data were converted into relative displacement vectors. This allowed the system to learn the displacement of the pen tip from its original location after movements of the pen, instead of the absolute coordinates. To this end, the difference between the data points among each sample was calculated, adding a zero in the first position to keep the same length of each sample. Additionally, this served as an alternative to data scaling since the absolute values of the data, on a high resolution scale, were reduced to the relative differences between the timesteps. Figure 5 shows an example of the character *’B’* in the absolute trajectory and the calculated relative displacement vectors. Sensor data was normalized using the Z-score normalization and used in the normalized raw form as input.

Additionally, data was divided into equal chunks of length of ten timesteps, equivalent to 100 milliseconds, after stroke interpolation, defined as *‘Chunks’* in the rest of the paper. This aims to represent the system in real time in which the sensor data was live streamed to the tablet, and the trajectory was predicted for each of the ten timesteps independently of other chunks. Chunking was not applied to label-interpolated data, since the tablet chunks included wrong data relative to the sensor chunks due to the same problem as shown in Figure 4.

The final dataset consisted of single words, represented in both IMU and relative-displacement-vectors data of equal lengths: label upsampled, stroke upsampled, and chunked. Deep-learning models were trained from scratch using this dataset, without relying on pre-trained models for trajectory reconstructing or handwriting recognition.

### 3.3. Trajectory Modeling

One dimensional convolutional neural networks (CNNs) were trained for mapping sensor IMU data to tablet coordinate data in a regression task, using the mean squared error (MSE) loss. As inputs, we used the channels corresponding to the two accelerometers, the gyroscope, and the force channel, displayed in Figure 2. The magnetometer channels were discarded, since the usage of tablet closely to the pen can affect the quality of the magnetometer measurements.

The architecture used in this paper is described in full detail in Table 2: the input layer was followed by three convolutional layers, each of which was followed by batch normalization, to reduce the training time and achieve better results, and dropout, to avoid overfitting. The output of the last convolutional layer was passed to a time-distributed fully connected (FC) layer consisting of two units, representing the X and Y coordinates. The Z coordinate channel was also discarded as it was not needed for the final inference of trajectories on the tablet. The models were trained on a batch size of 64 samples, with a learning rate of 0.0001. Long short-term memory (LSTM) neural networks were not implemented, since the aim was to predict a time-distributed relative displacement, and, therefore, learning long-term dependencies was not required.

First, we tested the performance of our model on the ground-truth trajectory data collected with the tablet. A leave-one-user-out (L1UO) cross validation was implemented, in which a model was trained on five users and tested on the sixth user to ensure that the system works for unknown users in a user-independent form. Table 3 shows the number of samples in the different folds, each sample representing a single word recording, totalling 2108 words samples. Six different models were generated (from the L1UO cross validation) for each of the different types of the processing methods (Label, Stroke, Chunks) mentioned in Section 3.2, totalling 18 models.

To test the generalisation capability in a surface-independent setup, three final models were trained on all users together, for the different pre-processing methods, which were later used to generate trajectories from IMU data of words written on paper, as discussed in Section 3.4.

### 3.4. Recognition Modeling

To evaluate the effectiveness of the TR system on data written on regular paper, text recognition models were implemented to test the quality of the generated trajectories, since ground-truth trajectory data of on regular paper is not available. The dataset provided by [32] was used, which consisted of 27,961 words written on plain paper using the Digipen. The trajectories for all samples in the paper dataset were generated using the three final models trained using all the samples from the tablet dataset, as discussed in Section 3.3, producing three datasets of the paper-written data as predicted trajectories.

First, we trained a model on the raw data of IMUs for the word-recognition task. For this purpose, the convolutional long-short term deep neural network (CLDNN) model defined in [32] was implemented. Similarly, the CLDNN model was implemented for the different generated trajectory datasets, since it achieved the best results in terms of recognition using the raw IMU data from the recorded words, producing different models for each dataset.

The data consisted of multiple samples of word instances, and each sample was represented as time-series data of different lengths, including 13 channels. Therefore, the model input layer consisted of 13 units, for all the channels recorded by the IMU sensors. Three convolutional layers, two LSTM layers, and a single FC layer followed, using the connectionist temporal classification (CTC) [37] loss with a Softmax output layer. Hyper-parameters were set similar to previous works, and a five-fold cross validation in a user-independent split, shown in Table 4, was implemented.

To train a word-recognition model on trajectories (instead of the IMUs signal), we modified the input layer in the previous architecture to be able to feed the generated trajectory datasets. Now, X and Y time-series data was used as input, while the rest of the architecture parameters were left unchanged. A total of 20 models were trained for the 5 folds, 5 using the IMU data and 15 for each of the generated trajectories.

Additionally, the models with the best results from the different models trained using the generated trajectories were used to train a joint IMU-coordinate model, using both IMU and trajectories as inputs to a neural network consisting of two parallel CLDNN branches, concatenating the outputs of the final FC layers in both branches before the CTC output layer.

## 4. Results

The neural network models learned the relative displacement vectors of the trajectories, i.e., the displacement from the position in the previous time step. Therefore, to acquire the original trajectories of what was written, the cumulative sum of all previous displacement vectors was calculated, in order to reconstruct the original word and acquire the absolute coordinates. The output trajectories were normalized using min–max scaling to evaluate the predictions on a unit scale. In this section, we present our findings and experiment results in both tasks, i.e., trajectory reconstruction and text recognition.

### 4.1. Trajectory-Reconstruction Evaluation

Root mean squared error (RMSE) was calculated to evaluate the similarity of the IMU-reconstructed trajectories in comparison to the ground truth from the original ones, which showed the normalized Euclidean-distance error between the ground truth and the prediction on the two dimensional plane.

Table 5 shows the results achieved for the different models over the different users. The L1UO cross validation on the models trained using the label-based interpolation resulted in a mean of 0.1864 normalized error on the unit scale. Stroke interpolation of the data showed an improvement in the performance of 5.47% in comparison to the label interpolation, achieving an error rate of 0.1762. Chunking the stroke-interpolated data showed similar results, with an average error of 0.1772, outperforming the un-chunked data in three of the six folds.

### 4.2. Text-Recognition Evaluation

Since the dataset consisted of only words, the character error rate (CER) was calculated to evaluate both the original IMU data and the generated trajectory data over the five folds. CER is calculated as:

(1) CER=(S+D+I)/N

where *S*, *D*, and *I* represent the substitutions, deletions, and insertions, respectively, required to obtain the correct word from a wrong sequence of characters. *N* represents the total number of characters in the given correct word. Table 6 shows the CER achieved of the different models over the five folds. The model trained using the IMU data resulted in an average CER of 16.0683%, and was the baseline to indirectly assess the quality of the generated trajectories.

The models trained using label-based interpolated data achieved the higher CER of 21.0789% on average, while stroke interpolation improved the CER by 1.2113%, with an average of 19.8676% error rate. Chunking the data slightly outperformed the un-chunked data, resulting in a 19.5103% CER.

The joint model, which uses both the IMU data and the generated trajectories as input, achieved an improved recognition rate of 15.3141% CER, outperforming the baseline IMU model by 0.7542%.

## 5. Discussion

The aim of this study was to develop a system that traces the movements of a sensor-enhanced digital pen and recreates written words on paper in a digital form displayed on a handheld device. Training data was collected by a digital pen on a tablet by multiple users, in a regular writing environment, and was used to develop a CNN-based system that works without using specific writing surfaces. The system was evaluated on tablet-written data using distance metrics by calculating the deviation in the predicted trajectories relative to the original recorded trajectories. Evaluation of paper-written data was carried out using a text-recognition system, rating the effectiveness of the system compared to a baseline model using IMU data.

### 5.1. Trajectory Reconstruction

CNNs were utilized to learn a time-distributed displacement of the pen tip, using sensor IMU recordings, for each timestep in the IMU data. However, due to the different sampling rates between the pen and tablet systems, the number of timesteps for a single sample recording differ with a ratio of 60 datapoints per second of coordinate data to 100 of sensor data. To acquire a similar length of timesteps, data acquired from the tablet were upsampled with linear interpolation to the length of the sensor data for each sample. Three different pre-processing methods were considered in our study to prepare the data for the neural network modeling of the trajectories, namely: label interpolation, stroke interpolation, and chunking.

#### 5.1.1. Pre-Processing Differences

**Figure 6:**
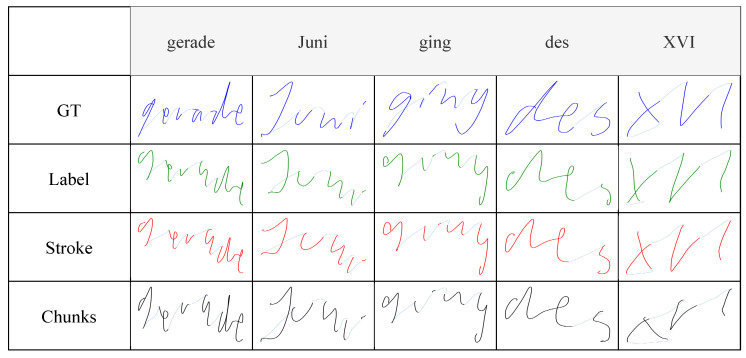
Trajectory recovery results when writing on tablet, including the ground truth trajectory collected by the tablet.

**Figure 7:**
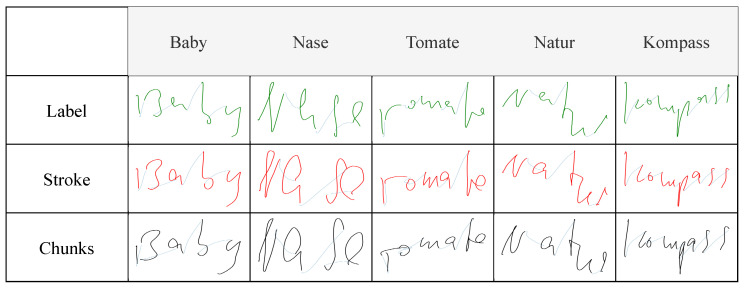
Trajectory recovery results when writing on paper.

Figure 6 shows the generated trajectories of random samples taken from the tablet dataset, displaying the ground-truth trajectories recorded on the tablet, as well as the trajectories predicted, by the different models, from the relative IMU data recorded by the pen. Generating trajectories from paper-written data showed similar results to tablet-written data, demonstrating that the system worked irrespective of the writing surface, as shown in Figure 7. Since trajectory GT data is not available for paper-written data, the system was also evaluated in a text-recognition application, discussed in Section 5.2.

A qualitative assessment of the generated trajectories showed that the model trained with label-interpolated data was able to learn the general movement of the pen but generated straight trajectories more than curved ones during inference. Examples can be seen in Figure 7, in which the curves in the letters *‘B’* and *‘s’* were clearer in the stroke-interpolated predictions. The reason behind this can be explained by Figure 4, where the four hovering strokes, having pressure equal to zero, in the label interpolation were longer than the hovering shown by the original IMU data, or the stroke-interpolated coordinate data. Since label interpolation upsampled the time series irrelevant of touch and hover strokes, hovering datapoints (which are mostly straight) were extended and unevenly distributed to the data relevant to touch datapoints from the original IMU data. Moreover, instances of writing were displayed as hovering, and vice versa, due to the same issue of having hover datapoints relative to writing movements of the pen. Stroke interpolation tackled these issues and generated smoother curved trajectories. Both presented similar results overall with slightly lower error rates presented by the model trained using stroke interpolated data. Though the differences are minimal in this study, the error differences will clearly be visible when considering longer recordings due to error accumulation, such as sentences, which is the final aim of the current study. Chunking the data and modeling every ten timesteps in the IMU data to the relative datapoints in the coordinate data showed similar results, implying that the system was able to learn specific short movements, independent of the the rest of the movements in a specific sample. This allowed the system to generate the trajectories from the IMU data every 100 milliseconds during a live-streamed writing session.

In terms of error rates, all models showed much lower error rates on the training data in comparison to the test data, reaching an average normalized error of 0.0484 compared to 0.1799, respectively, implying that the models still overfitted, and can be improved to achieve lower error rates if more data was available in future works. Finally, even with adequate results achieved by CNNs, different architectures can be considered, such as Autoencoders, that have shown acceptable results in retracing trajectories from text images [38,39,40].

#### 5.1.2. Data Limitations

Though the models achieved notable results and quality trajectories, some drawbacks were identified in a testing session of the system. Characters in a retraced word did not always align correctly in a straight writing line as originally written. This was due to having higher error rates in hovering strokes in comparison to touch strokes, on average reaching 0.1834 and 0.1581, respectively. This occured as a result of having different ratios between touch and hover data, as the written words in the dataset consisted more of writing strokes with fewer hovering strokes occurring between characters, or between multiple strokes of single characters. The ratio of touch to hover timesteps in the dataset was approximately 68% to 32%, respectively, calculated by the average length of the strokes among the different samples. Since the nature of the IMU data differs when writing, in contrast to hovering, due to surface friction, the trained models achieved inferior results on hover data between the different strokes, since touch data is more prevalent in the used dataset.

The developed system was completely data-driven. Consequently, the quality of the data used for training the models is significant, and, as such, any available bias in the data affects the final output of the system. The collected data consisted of written words, in the Latin alphabet, and written from left to right. Therefore, the models learned constant movement to the right even when there was none, which was seen when hovering the pen without movement while testing. Examples can be seen in Figure 6 and Figure 7, in which the second stroke of the letters *’i’* and *’K’* were further to the right of the first stroke of the letters instead of on top.

To avoid such limitations of the system in future studies, the data-collection process has to be adapted to include more hovering data while ensuring a balance between touch and hover strokes within the dataset. Similarly, the constant right movement of the predicted trajectories can also be considered within the data-collection process, adapting the labels to different movements instead of regular words, ensuring the data includes a wide variance in movements in different directions over the tablet.

### 5.2. Text Recognition

A paper-written word dataset was provided by previous works to test the effectiveness of the generated trajectories by our models in a word-recognition system. Additionally, the models implemented in this study were inspired by the model that achieved the best results on the mentioned dataset. Several models were implemented for the different pre-processing steps and evaluated separately.

Similar to the results presented by the trajectory models, the recognition model using stroke-interpolated data achieved better results than the model trained with label interpolated data, while chunked data also produced similar results to the un-chunked data. Overall, the models using generated trajectory data achieved inferior results compared to the models trained using the IMU data; however, its performance is promising: more trajectory data and lower error rates in the trajectory modeling could lead to better recognition rates.

The joint models using both IMU and chunk data as inputs showed the best results, outperforming the baseline model trained with the IMU data only, implying that with better reconstruction results, it is possible to improve general recognition algorithms using IMU data recorded by the Digipen.

### 5.3. Comparison to State-of-the-Art Approaches

The system presented in this paper surpassed previously developed solutions for application in handwriting reconstruction in terms of word reconstruction. With regards to data pre-processing and preparation, previous solutions required multiple initialization steps, such as segmenting recordings into writing and hovering, calculating the displacement of a pen tip relative to a global coordinate system, and applying rotation matrices to estimate the final displacement of the pen. In terms of reconstruction, each segmented stroke was reconstructed, then the estimated tip positions were connected chronologically. Finally, angle calculation and orientation was needed to align the recovered trajectory to the initial readable format. Moreover, the recognition systems presented were for single-character recognition only.

The presented solution was developed with minimal pre-processing, requiring only the stroke interpolation of the coordinate data to acquire time-distributed datapoints. To the knowledge of the authors, it is the first data-driven, neural network based system that efficiently recovers handwriting in real time. Additionally, no post-processing alignment was required to recover the correct pen tip displacement in a correct readable format. The models required no data segmentation, but instead, learned the hovering movements of the pen in addition to the writing movements, which was a limitation in previous works in which the retraced trajectories were mostly for continuous single strokes. In terms of efficiency, the absence of conventional pre-processing methods, and post-processing alignment methods, reduced the complexity of the system averaging an inference time of 0.052 s, relative to previously developed models with a prediction time reaching up to 4 s. Moreover, since the data was collected in regular writing conditions, no situation-specific adaptations of the system are required. This paper also presented a word recognition model of the reconstructed trajectories, and was not limited to single stroke reconstruction or single letter recognition. However, concerning general image reconstruction, the system still lagged behind in recovering the trajectories of random images written on paper due to data limitations discussed in Section 5.1.2.

## 6. Conclusions

The goal of this work was to develop a system capable of reconstructing handwriting on paper into a digitized form on a handheld device. Data was collected from six participants who wrote random words on a tablet using a digital pen equipped with several IMU sensors. Three pre-processing methods were introduced to prepare the data for neural network modeling, and test the effectiveness of the system for real-time trajectory recovery. CNNs were implemented to map the recorded sensor data to the relative trajectory coordinate data using a supervised learning approach. A L1UO cross validation resulted in a mean normalized error of 0.1762 on a unit-scale tablet trajectory evaluation.

The developed models were used to recover images from paper-written data, provided by a previously used dataset [32] for the development of a word recognition system. Generated trajectories were then used to develop similar recognition models previously introduced, achieving a character recognition rate of 80.4897% for word recognition. Moreover, a joint model using both IMU data and reconstructed trajectory data was implemented, outperforming IMU-only models, reaching a character recognition rate of 84.6859%.

In summary, handwriting TR is a challenging problem due to the small surface area upon which writing takes place, yet is a significant application due the importance of handwriting in daily lives. Current solutions demand the usage of specific writing surfaces, detecting the tip of digital pens on the relative writing surfaces. This paper presented a surface-free solution for reconstructing handwritten words. We demonstrated effectiveness of our method on reconstructing trajectories on a small scale, without relying on classical sensor fusion methods, but instead following a fully data-driven approach.

Future work following this would include the reconstruction of complete sentences without limiting the system only to words. Moreover, the system would be generalized for random image reconstruction, without limiting the system to the Latin alphabet, which allows the development of a multi-lingual trajectory reconstruction system that is not solely based on the Latin alphabet.

## Figures and Tables

**Table 1:** Summary of the related work of trajectory reconstruction systems. *Gyr* and *Acc* represent gyroscope and accelerometer, repectively.

Authors	Strokes	Segmentation	Calibration	TR Method	Recognition Method	Recognition Type
[17]	Single	-	Yes	Gyr single integration	Apple Newton recognizer	Words
[33]	Single	-	Yes	Acc double integration	Hidden markov models	Characters
[18]	Single	-	Yes	Acc double integration	Hidden markov models	Characters
[34]	Single	-	Yes	Acc double integration	Vista tablet PC recognizer	Digits
[19]	Multi	Yes	Yes	Acc double integration	Google IME recognizer	Characters
Proposed Model	Multi	No	No	Neural networks	Neural networks	Words

**Table 2:** Detailed description of the convolutional neural network used in this study.

Layer	Hyperparameters	# of Parameters
1D convolution	Filters: 256, Kernel-size: 3	7936
Batch normalization	Momentum: 0.99, Epsilon = 0.001	1024
Dropout	Rate: 0.3	0
1D convolution	Filters: 256, Kernel-size: 3	196,864
Batch normalization	Momentum: 0.99, Epsilon = 0.001	1024
Dropout	Rate: 0.3	0
1D convolution	Filters: 256, Kernel-size: 3	196,864
Batch normalization	Momentum:0.99, Epsilon = 0.001	1024
Dropout	Rate: 0.3	0
TimeDistributed (fully connected)	Units: 2	514
**Total parameters**		405,250

**Table 3:** User-tablet data recordings split into leave-one-user-out folds.

Folds	# of Training Samples	# of Test Samples
1	1774	334
2	1608	500
3	1941	167
4	1718	390
5	1875	233
6	1624	484

**Table 4:** User paper data recordings split into five user-independent folds.

Folds	# of Training Samples	# of Test Samples
1	24,087	3874
2	22,877	5084
3	22,896	5065
4	22,900	5061
5	22,896	5065

**Table 5:** Normalized TR error rates over the different users.

	1	2	3	4	5	6	Mean
Label	0.1649	**0.2149**	0.1734	0.1928	0.1764	0.1964	0.1864
Stroke	0.1633	0.2154	**0.1615**	0.1634	0.1726	**0.1812**	**0.1762**
Chunks	**0.1608**	0.2285	0.1654	**0.1628**	**0.1621**	0.1838	0.1772

**Table 6:** Character error rates over the five folds of the paper data using IMU data and generated trajectories.

	1	2	3	4	5	Mean
IMU	14.9484	27.3908	11.5514	12.9280	13.5233	16.0683
Label	20.7685	30.1311	19.4653	18.8399	16.1897	21.0789
Stroke	20.3729	28.7688	**17.1135**	18.1958	14.8872	19.8676
Chunks	**20.0750**	**28.5405**	17.3706	**17.4865**	**14.0788**	**19.5103**
Joint IMU-Chunks	15.2440	25.3947	11.2754	12.3797	12.2769	15.3141

## Data Availability

The data is available from the authors upon request, and is also planned to be published as a larger dataset including character, sentence, and random drawing recordings.

## Abbreviations

The following abbreviations are used in this manuscript:

TR	Trajectory reconstruction
IMU	Inertial measure unit
CNN	Convolutional Neural Network
GT	Ground truth
LSTM	Long short-term memory
L1UO	Leave-one-user-out
FC	Fully connected
CLDNN	Convolutional long-short term deep neural network
CER	Character error rate
